# Comparison of Fc *N*-Glycosylation of Pharmaceutical Products of Intravenous Immunoglobulin G

**DOI:** 10.1371/journal.pone.0139828

**Published:** 2015-10-12

**Authors:** Willem Jan R. Fokkink, David Falck, Tom C. M. Santbergen, Ruth Huizinga, Manfred Wuhrer, Bart C. Jacobs

**Affiliations:** 1 Department of Immunology, Erasmus MC, University Medical Center Rotterdam, Rotterdam, The Netherlands; 2 Department of Neurology, Erasmus MC, University Medical Center Rotterdam, Rotterdam, The Netherlands; 3 Center for Proteomics and Metabolomics, Leiden University Medical Center, Leiden, The Netherlands; 4 Division of BioAnalytical Chemistry, Department of Chemistry and Pharmaceutical Sciences, Division of BioAnalytical Chemistry, VU University, Amsterdam, The Netherlands; University of Würzburg, GERMANY

## Abstract

Intravenous immunoglobulin (IVIg) products from different pharmaceutical companies vary in composition, in part because of the selected blood donors and production process. *N*-glycosylation of the Fc-portion of IgG varies between blood donors and may influence both the side-effects and therapeutic effectiveness of IVIg. At present, the variation in Fc *N*-glycosylation between IVIg products has not been defined. Utilizing mass spectrometry, we performed relative quantitation of the Fc *N*-glycosylation of IgG, assessing a total of 154 unique lot numbers of IVIg. Seven products showed comparable Fc *N*-glycosylation, with only one product differing from the others in all glycosylation features (galactosylation, sialylation, fucosylation and bisecting *N*-acetylglucosamine). However, the mean difference did not exceed 3%. Within product variation was present to a minor degree, but largely indistinguishable from analytical variation. In conclusion, we expect that the minor variation in Fc *N*-glycosylation between IVIg products has a small effect, if any, on the biological activity.

## Introduction

Intravenous immunoglobulin (IVIg) is the mainstay treatment for a broad spectrum of immune deficiencies and autoimmune diseases. In addition to the FDA approved indications, IVIg is frequently used off-label for other (immune) disorders [1,2]. For years the world consumption and costs of IVIg has been increasing, creating a billion dollar global market [3,4]. Only a select number of pharmaceutical companies (and local blood banks) market IVIg, and these products differ with respect to the blood donor pool and production methods, leading to variation in the product composition (e.g. IgG subclass distribution, stabilizers, sodium, IgA) [5–8]. However, only a few studies have compared the clinical efficacy of different IVIg products used for high-dose immune-modulatory treatment [9]. Some studies found no differences between the IVIg products, while others reported a discrepancy in the occurrence of side-effects, patient tolerability and even outcome [10–14].

Despite the extensive use of IVIg, remarkably little is known about the working mechanism, pharmacokinetics, optimal dosages and regimens [1,15]. Multiple postulated working mechanisms have been explored in animal models and human studies [16]. One of the more recent proposed modes of action depends on the *N*-glycosylation of the Fc portion of the IgG [17–20]. Some studies indicate that the therapeutic effect of IVIg is based on a minor fraction of IgG, with a specific glycoform in IVIg, implicating that enrichment of these IgG glycoforms in IVIg might potentially lead to a more effective product [17–19,21]. While the WHO has formulated minimum requirements for the production of IVIg for clinical use, monitoring of the IgG glycosylation, to our knowledge, is not among them [22,23]. There are at present no comparative studies published on the *N*-glycosylation of the Fc-part of IgG in IVIg, which is known to be influenced by age, gender and the environment [24]. Therefore, since the population of blood donors differs between products [25,26], the glycosylation of IVIg products may be influenced.

In the current study, we determined the variation in IgG Fc *N*-glycosylation between different batches and brands of IVIg products available on the Western-European market for therapeutic use.

## Results

### Inter-products differences

**Fig 1:**
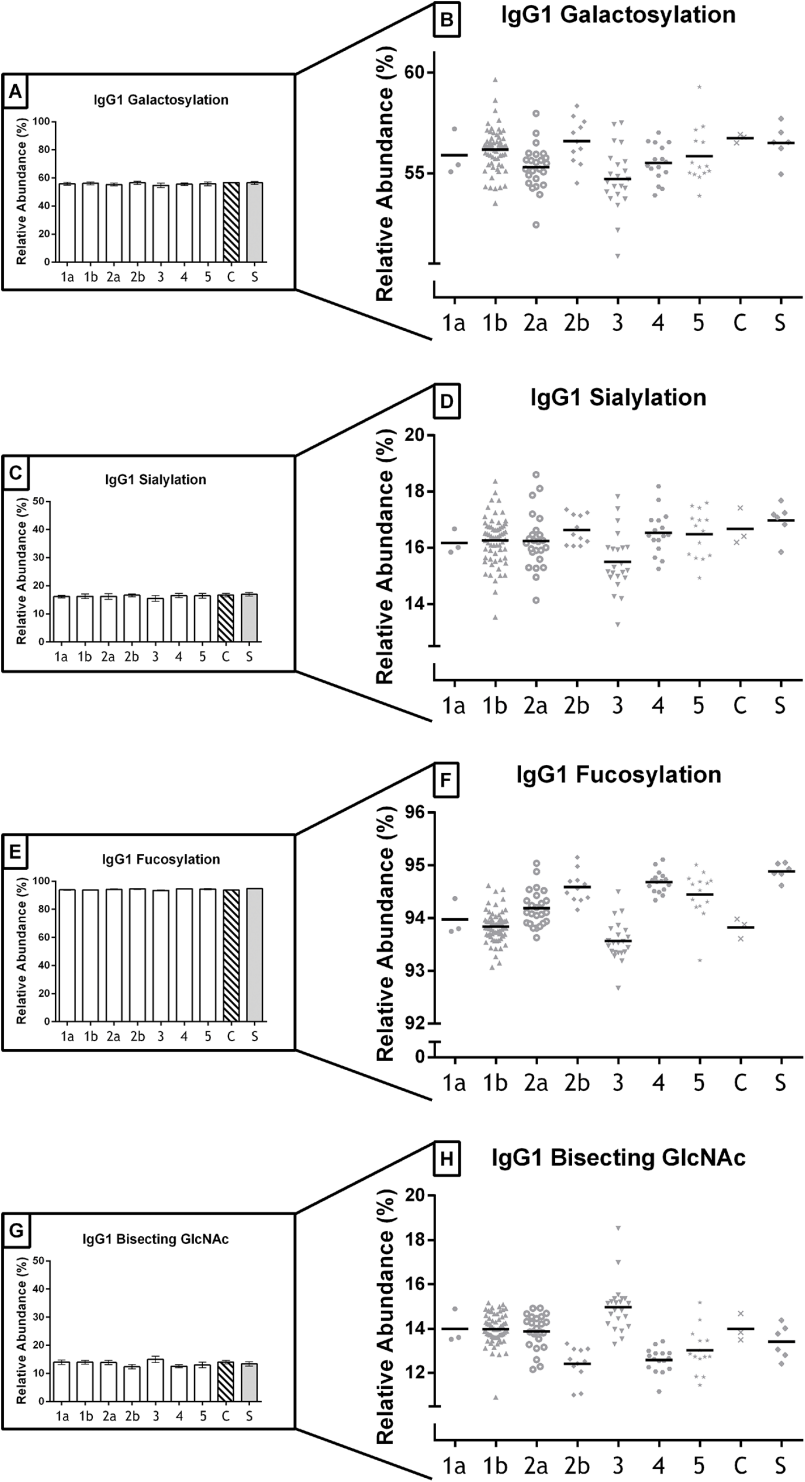
IgG Fc-glycosylation of IVIg preparations available on the Western-European market for therapeutic use. In total 154 unique IVIg batches produced by 5 different companies (1 to 5) were analyzed, consisting of 7 products (1a n = 3, 1b n = 64, 2a n = 24, 2b n = 11, 3 n = 22, 4 n = 16, 5 n = 14, with the capital S denoting the IgG standard (n = 6) and the capital C denoting an IVIg triplicate of the same batch). Galactosylation for IgG1 presented as A) mean (SD) per product, and B) individual results for all tested batches per product (bold line denoting the median). The same is shown for the other glycosylation features; C and D for sialylation, E and F for fucosylation and G and H for bisecting GlcNAc.

The normalized relative intensities of the IgG1 Fc *N*-galactosylation. sialylation, fucosylation and bisecting *N*-acetylglucosamine (GlcNAc) for all analyzed IVIg products and controls are presented in Table 1 and Fig 1. Afucosylated species for IgG2/3 were below the limit of detection. In preparations #3 the mean galactosylation of IgG1 was slightly lower than in the other products (3 vs 1B and 2B, *P* <0.001). The same was found for this IVIg preparation in IgG1 sialylation and fucosylation and IgG2/3 galactosylation and sialylation, with significantly lower levels than some of the other samples (ANOVA with post-hoc Tukey Test, S1 Table). Conversely the bisecting GlcNAc was significantly increased in preparation #3 (for IgG1 3 vs 1B, *P* = 0.005, and for 3 vs 2B, 4, 5 *P* <0.001). Correlations between galactosylation and sialylation within the respective subclasses were strong (IgG1 *r* = 0.804, IgG2/3 *r* = 0.909, *P* < 0.001 for both), with no significant difference between the correlation coefficients of the tested products (Fisher r-to-z transformation for correlation coefficients). The same was found for the correlation between fucosylation and bisecting GlcNAc (IgG1 *r* = -0.844, *P* <0.001). While the minor serum subclasses (IgG2/3) overall gave comparable patterns to IgG1 with respect to galactosylation, sialylation and bisecting GlcNAc, absolute levels differ (e.g. a lower absolute level of galactosylation for IgG2/3, Table 1 and S1 Fig); also there was only a weak to moderate correlation between the subclasses (galactosylation IgG1 –IgG2/3 *r* = 0.217, *P* = 0.007; sialylation IgG1 –IgG2/3 *r* = 0.235, *P* = 0.003; and bisecting GlcNAc IgG1 –IgG2/3 r = 0.545, P <0.001).

**Table 1:** Overview of the IgG Fc *N*-glycosylation of seven different IVIg products.

		1A	1B	2A	2B	3	4	5	C	S
		(n = 3)	(n = 64)	(n = 24)	(n = 11)	(n = 22)	(n = 16)	(n = 14)	(n = 3)	(n = 6)
IgG1	Mean	55.90	56.17	55.30	56.59	54.72	55.51	55.85	56.75	56.5
Galactosylation	SD	1.14	1.05	1.10	1.15	1.56	0.92	1.39	0.21	0.92
	CV (%)	2.04	1.87	1.98	2.03	2.85	1.65	2.49	0.38	1.63
IgG1	Mean	16.17	16.26	16.24	16.63	15.50	16.53	16.48	16.67	16.98
Sialylation	SD	0.44	0.84	1.01	0.52	1.06	0.77	0.84	0.66	0.62
	CV (%)	2.71	5.19	6.20	3.13	6.84	4.63	5.09	3.93	3.64
IgG1	Mean	93.97	93.84	94.19	94.59	93.57	94.68	94.45	93.82	94.89
Fucosylation	SD	0.34	0.30	0.35	0.29	0.37	0.20	0.45	0.19	0.16
	CV (%)	0.37	0.32	0.3	0.31	0.4	0.22	0.48	0.2	0.17
IgG1	Mean	14	13.97	13.88	12.41	14.97	12.58	13.01	14	13.41
Bisecting	SD	0.77	0.69	0.78	0.78	1.12	0.55	1.01	0.61	0.76
GlcNAc	CV (%)	5.49	4.97	5.62	6.28	7.51	4.39	7.75	4.38	5.68
IgG2/3	Mean	49.41	46.88	46.43	48.09	46.09	47.85	47.65	47.77	46.63
Galactose	SD	0.82	0.20	0.31	0.65	0.32	0.35	0.28	0.52	0.56
	CV (%)	2.87	3.43	3.31	4.49	3.23	2.92	2.23	1.88	2.93
IgG2/3	Mean	18.12	16.73	17.03	17.41	16.09	17.93	17.52	17.39	16.61
Sialic acid	SD	0.54	0.13	0.24	0.40	0.26	0.28	0.22	0.23	0.49
	CV (%)	5.21	6.28	6.92	7.62	7.53	6.17	4.66	2.27	7.21
IgG2/3	Mean	11.44	11.53	11.65	10.85	11.97	10.88	11.07	11.47	11.77
Bisecting	SD	0.43	0.53	0.47	0.57	0.35	0.35	0.43	0.31	0.32
GlcNAc	CV (%)	3.75	4.63	4.04	5.24	2.92	3.24	3.87	2.67	2.73

Data presented as mean, standard deviation (SD) and coefficient of variation (CV), numbers in the first row are the seven different IVIg products, (C) denotes an IVIg batch triplicate, and (S) an internal IgG standard.

### Intra-products differences

Notably, the standard deviations for Fc-glycosylation did not differ significantly for the different products (Brown-Forsythe test) or when compared to an IVIg control sample (i.e. accounting for analytical variation, F-test). Hence, it appeared to be similar in size or smaller than the analytical variation. The observed variation was largest for preparation #1B IgG1, yet this may for a large part be caused by analytical variation (Table 1). Incorporating outliers in preparation #1B yields a variation in IgG1 galactosylation of 53.5–59.7% (range, min–max), 13.5–18.4% for IgG1 sialylation, 43.3–52.5% for IgG2/3 galactosylation, and 8.75–12.33% for IgG2/3 bisecting GlcNAc. Preparation #4 showed the largest variation for IgG1 Bisecting GlcNAc of 13.30–18.52% (range, min–max), for fucosylation of 92.67–94.50% and for IgG2/3 sialylation of 16.7–21.5%.

## Discussion

This study showed that the seven IVIg products commonly used for treatment of patients in The Netherlands have a similar IgG Fc *N*-glycosylation. Although some preparations showed differences in mean IgG Fc galactosylation, sialylation, fucosylation and/or bisecting GlcNAc, the absolute mean differences were not in excess of 3% apart from one preparation (#3). Nonetheless, changes in fucosylation can have profound influence on antibody function [27]. But for galactosylation, for example, these differences are within the range of natural variation of IgG glycosylation between sexes as well as between individuals in general, and are smaller than age-related changes associated with an age difference of a decade [28]. Notably, the differences between IVIg preparations are approximately 1 order of magnitude smaller than the IgG glycosylation changes observed with major inflammatory conditions such as rheumatoid arthritis and osteoarthritis [29]. Since the spread in the control samples is comparable to those in the IVIg products, within batch variation could mostly be attributed to analytical variation.

It was previously demonstrated that the LC–MS technique employed in this study is suitable for the detection of minor, functionally relevant differences in IgG Fc *N*-glycosylation [30]. The quality of the data obtained for the samples as well as positive and negative controls, confirm the suitability of the platform for the analysis of biopharmaceutical formulations. In addition, we did not find any obvious analytical biases: there was no significant correlation, neither per sample nor per formulation, of the different glycosylation trades with spectral intensity or position in the analysis sample queue (and consequently the plate position). Thus, it is likely that the observed differences in glycosylation are actual product differences. A caveat to be made is the unequal number of samples available for analysis of each preparation. Also, the products analyzed were derived from the Western-European market. Therefore, we cannot extrapolate our findings to all IVIg markets.

In the current study, we have focused on the main categories of glycosylation instead of individual glycoforms, although specific glycoforms may differ between individuals and influence the effects of IgG [31,32]. A study in patients with multiple myeloma has shown that each IgG paraprotein in serum may exhibit a unique oligosaccharide profile [31]. The glycosylation of IgG appeared to be influenced by the local environment since the polyclonal IgG in serum from these patients may reflect the glycoform of the IgG paraproteins. IVIg as a polyvalent product pooled from (ten) thousands of healthy individuals, is expected to contain the full range of known human IgG Fc-glycoforms in healthy blood donors. With the technique used in the current study we were able to assign unambiguously 26 separate IgG glycoforms. These glycoforms may differ substantially between the individual blood donors, in part influenced by demographic factors (e.g. age of the donor), but the sheer size of the mixed donor pool of IgG in IVIg likely masks these inter-individual differences and contributes to the similar glycoform distribution observed in the IVIg preparations. In addition, the impact of individual glycoforms on IgG function is, hitherto, largely unknown, and therefore we focused on derived glycosylation traits.

To our knowledge no other extensive studies were published on the variation in Fc *N*-glycosylation of IVIg. However, the results presented here can be compared to the serum IgG Fc *N*-glycosylation of healthy controls (which IVIg is derived from, albeit as a mixture of thousands of healthy donors), measured previously by our group with the same technique [30]. In general, the mean galactosylation, sialylation and fucosylation of all IVIg preparations combined appear slightly higher than in healthy controls (IgG1 mean galactosylation 55.8% (SD 1.8), IgG1 sialylation 16.2% (SD 0.9) and IgG1 fucosylation 94,0% (SD 0.5) in this work, compared to that of 91 previously tested healthy controls of 53.4% (SD 8.1), 14.5% SD (3.7) and 93.0% (SD 3.0), respectively). The level of bisecting GlcNAc was lower in the IVIg preparations (for IgG1 13.8%, SD 1.1) than in the previously tested serum IgG from healthy controls (19.4%, SD 4.0). These differences may be due to a possible lower mean age of the blood donors used to produce IVIg compared to the healthy control group used in the previous study [30]. Note, the relatively small standard deviations of IVIg relative to the healthy controls as well as to the other studies mentioned earlier [28,29]. Thus, intra- and inter-product variation are, in any case, significantly lower than normal biological variation. The correlations between galactosylation–sialylation and fucosylation–bisecting GlcNAc in IVIg were similar to that of IgG directly obtained from human serum, as described previously [24,33]. The sialylation, deemed important for the anti-inflammatory effects, appeared not to be greatly influenced by the IVIg manufacturing process. Some studies showed that the anti-inflammatory effects of Fc-sialylation in IVIg may not occur in all disease models, indicating that IVIg may have different modes of action in different diseases [34,35]. For further comparison, a previous study, assessing therapeutic monoclonal antibodies (IgG based recombinant production), found acceptable intra-product variation in Fc-glycosylation [36]. It is therefore likely that the even smaller variations in Fc *N*-glycosylation for IVIg reported here are also acceptable.

In conclusion there are small differences between IVIg products with respect to the functionally relevant Fc-*N*-glycosylation of IgG. We expect that these minor differences will only have a small or no effect on the biological activity of the IVIg preparations.

## Materials and Methods

In total 154 unique samples were collected of seven different IVIg products from five pharmaceutical companies (#1–5, with 1a/b and 2a/b representing different products produced by the same manufacturer, for lot-numbers see S2 Table). In addition, three replicates of a single IVIg sample and an IgG standard (Human Plasma Immunoglobulin G, Athens Research and Technology) were included in the analysis in order to assess analytical variation. Small volume aliquots from the remaining IVIg in the flask after infusion were stored at -80°C until use. The majority of samples were collected from December 2012 until September 2013, complemented by additional samples collected prior to this period. Fc *N*-glycosylation was measured by mass spectrometric analysis of glycopeptides according to Selman *et al*. [37], but without the protein A affinity purification step, which was not required since all IVIg products consisted of >95% IgG. Partial denaturation of IgG was achieved by 15 min incubation at room temperature in 75 mM formic acid followed by drying via vacuum centrifugation. Thereafter, IVIg samples were reconstituted in 40 μl 50 mM ammonium bicarbonate (pH 8.0) containing 2 μg TPCK-treated trypsin (Sigma-Aldrich, Schnelldorf, Germany). After approximately 18 h of tryptic digestion, IgG Fc glycopeptides were analysed by liquid chromatography–mass spectrometry (LC–MS). IgG subclasses 1 and 4 yield unique glycopeptides while subclasses 2 and 3 share the same peptide backbone for the glycopeptides preventing there distinction by LC–MS [38]. Therefore, summed profiles are given for IgG2 and 3. The method used can even account for potential IgG allotypic variation within the glycopeptide sequence [39,40]. IgG4 was excluded as the low abundance of its glycopeptides prevented an analysis of sufficient quality. Normalization to the sum of glycopeptide intensities per subclass yielded relative intensity profiles from which the levels of glycosylation features (galactosylation, sialylation, fucosylation and incidence of bisecting GlcNAc) were calculated similarly to the previously described procedure [30]. However, due to different and varying sample concentrations, the extracted features and, consequently the derived trade calculations were different. For galactosylation, sialylation, fucosylation and bisecting GlcNAc, we used the following formulas, respectively: Galactosylation = 0.5*(G1F+G1FN+G1FS)+(0.5*G1)_IgG1only_+G2F+G2FN+G2FS+(G2+G2S)_IgG1only_; Sialylation = G1FS+G2FS+(G2S)_IgG1only_; Fucosylation = G0F+G1F+G2F+G0FN+G1FN+G2FN+G1FS+G2FS; Bisecting GlcNAc = G0FN+G1FN+G2FN. Glycosylation levels of the respective IVIg brands were compared using ANOVA and the post-hoc Tukey test (accounting for multiple testing). Variance within the batches was tested with the Brown-Forsythe test (group-wise) and the F-test compared to the variance of the three IVIg replicates (versus each product). Coefficient of variation (CV, defined as the standard deviation divided by the mean) was used to describe within-batch variation. Two-sided p-values <0.05 were considered to be statistically significant and all statistics were performed with GraphPad Prism (6.0) and SPSS statistics (21.0).

## Supporting Information

S1 Fig IgG Fc-glycosylation of IVIg preparations available of IgG2/3. Comparable to Fig 1, the 154 unique IVIg batches produced by 5 different companies (1 to 5) were analyzed, consisting of 7 products (1a n = 3, 1b n = 64, 2a n = 24, 2b n = 11, 3 n = 22, 4 n = 16, 5 n = 14, with the capital S denoting the IgG standard (n = 6) and the capital C denoting an IVIg triplicate of the same batch). Galactosylation for IgG1 presented as A) mean (SD) per product, and B) individual results for all tested batches per product (bold line denoting the median). The same is shown for the other glycosylation features; C and D for sialylation, E and F for bisecting GlcNAc.
(TIF)

S2 Fig Differences between IVIg preparations. (PDF)

S1 Table Differences between IVIg preparations. (DOCX)

S2 Table IVIg lot-numbers. (DOCX)

S3 Table Overview of all glycoforms for the tested IVIg preparations. (XLSX)
