# Supplementary figures and images for: Comparison of Fc N-Glycosylation of Pharmaceutical Products of Intravenous Immunoglobulin G
Source: PLoS One. 2015 Oct 12;10(10):e0139828. doi: 10.1371/journal.pone.0139828 (PMC4601728; doi:10.1371/journal.pone.0139828)

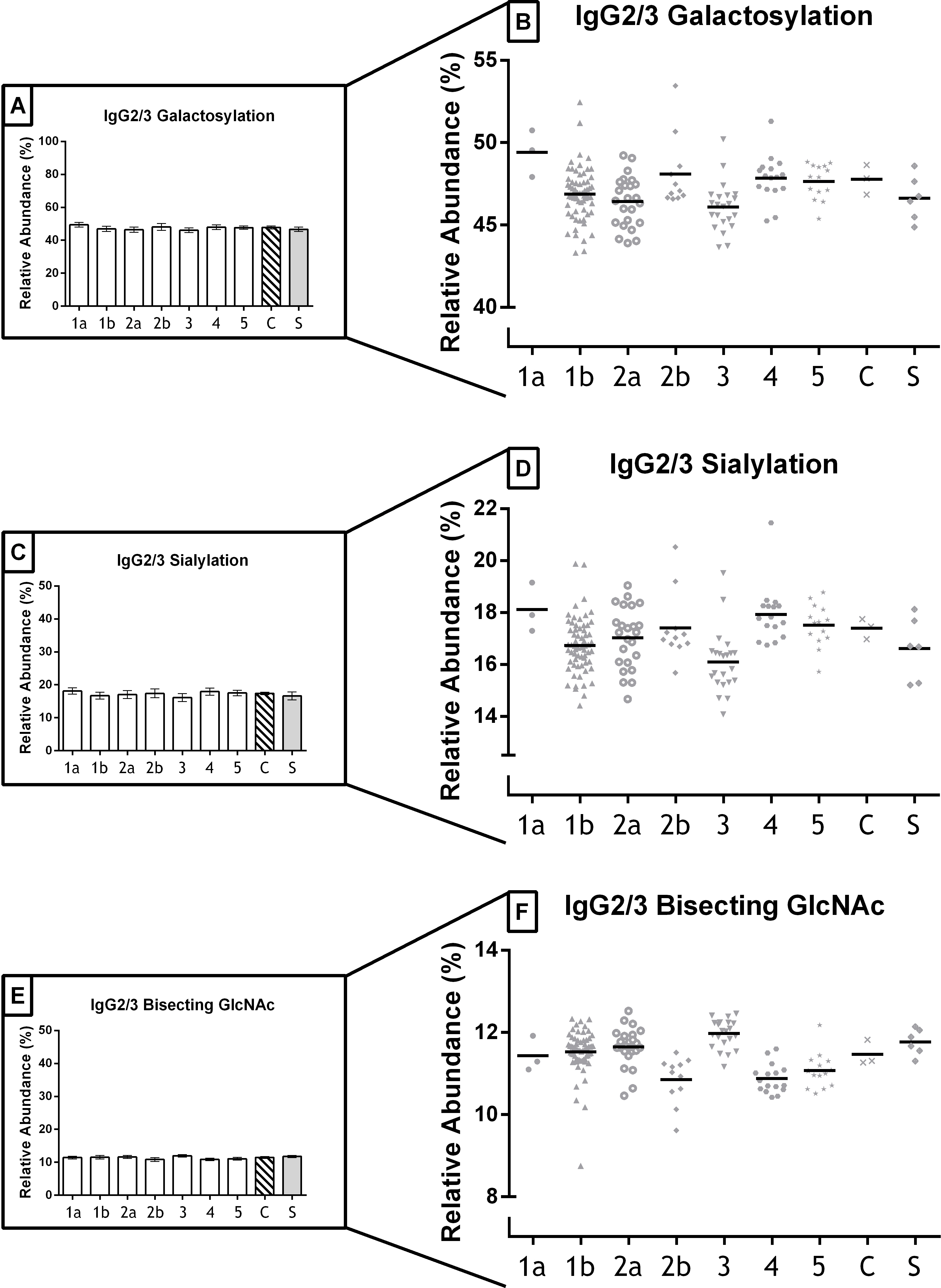

Supplement: S1 Fig — Comparable to Fig 1, the 154 unique IVIg batches produced by 5 different companies (1 to 5) were analyzed, consisting of 7 products (1a n = 3, 1b n = 64, 2a n = 24, 2b n = 11, 3 n = 22, 4 n = 16, 5 n = 14, with the capital S denoting the IgG standard (n = 6) and the capital C denoting an IVIg triplicate of the same batch). Galactosylation for IgG1 presented as A) mean (SD) per product, and B) individual results for all tested batches per product (bold line denoting the median). The same is shown for the other glycosylation features; C and D for sialylation, E and F for bisecting GlcNAc. (TIF) [file pone.0139828.s001.tif]
