# Supplementary material for: Comparison of Fc N-Glycosylation of Pharmaceutical Products of Intravenous Immunoglobulin G
Source: PLoS One. 2015 Oct 12;10(10):e0139828. doi: 10.1371/journal.pone.0139828 (PMC4601728; doi:10.1371/journal.pone.0139828)
Supplement: S2 Fig — (PDF) [file pone.0139828.s002.pdf]

Supplementary figure 2. Differences between IVIg preparations in respect of four glycosylation features for IgG1 (A-D) and three features for IgG2/3 (E-G). Tested with ANOVA and a post-hoc Tukey Test.

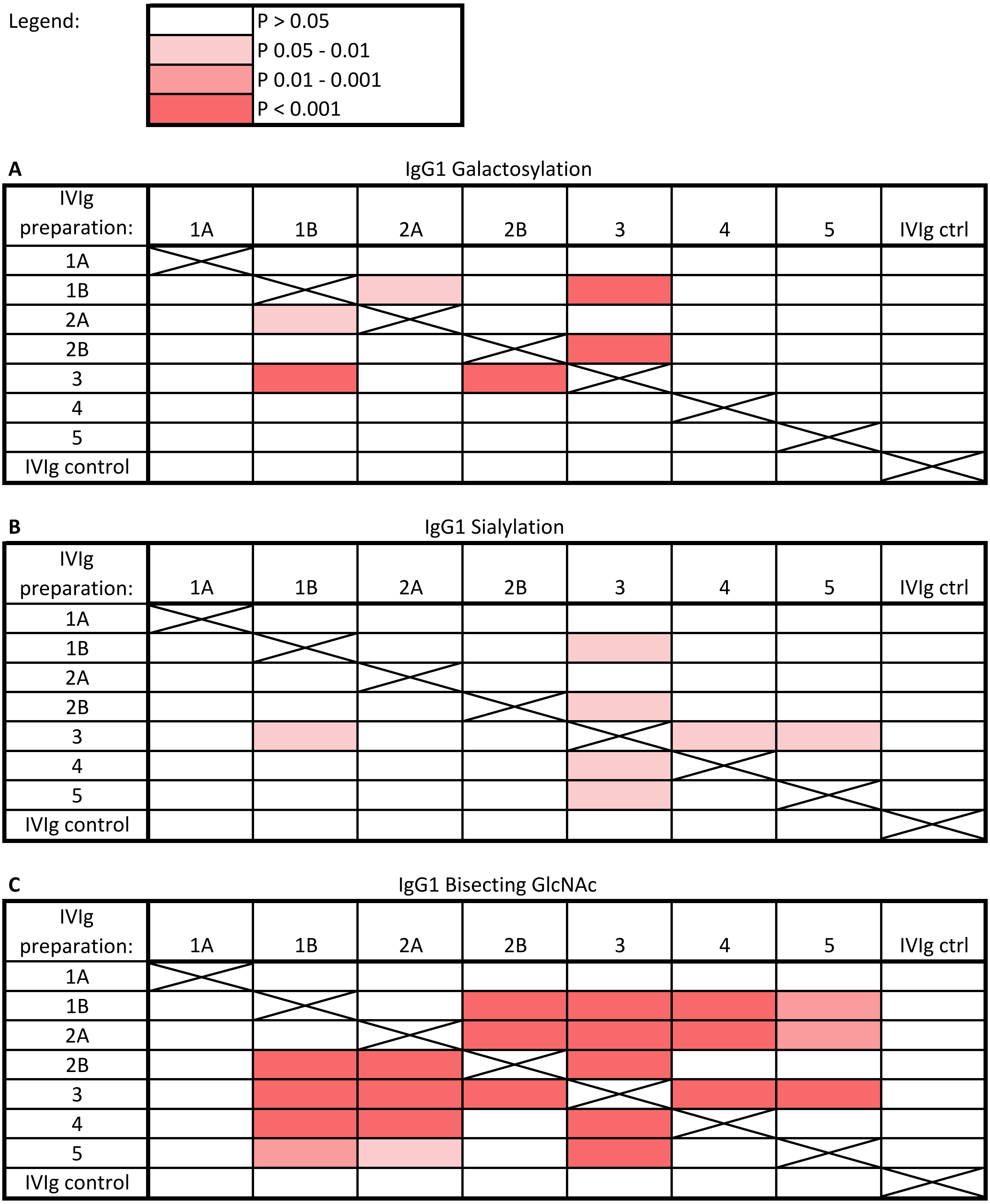

D IgG1 Fucosylation

IVIg preparation:	1A	1B	2A	2B	3	4	5	IVIg ctrl
1A								
1B								
2A								
2B								
3								
4								
5								
IVIg control								

E IgG2/3 Galactosylation

IVIg preparation:	1A	1B	2A	2B	3	4	5	IVIg ctrl
1A								
1B								
2A								
2B								
3								
4								
5								
IVIg control								

F IgG2/3 Sialylation

IVIg preparation:	1A	1B	2A	2B	3	4	5	IVIg ctrl
1A								
1B								
2A								
2B								
3								
4								
5								
IVIg control								

G IgG2/3 Bisecting GlcNAc

IVIg preparation:	1A	1B	2A	2B	3	4	5	IVIg ctrl
1A								
1B								
2A								
2B								
3								
4								
5								
IVIg control								
