# Supplementary material for: Comparison of Fc N-Glycosylation of Pharmaceutical Products of Intravenous Immunoglobulin G
Source: PLoS One. 2015 Oct 12;10(10):e0139828. doi: 10.1371/journal.pone.0139828 (PMC4601728; doi:10.1371/journal.pone.0139828)
Supplement: S1 Table — (DOCX) [file pone.0139828.s003.docx]

| Tukey's multiple comparisons test | Mean Diff, | 95% CI of diff, | Adjusted P Value |
| --- | --- | --- | --- |
|  |  |  |  |
| 1A vs. 1B | -0,2751 | -2,391 to 1,841 | > 0,9999 |
| 1A vs. 2A | 0,5979 | -1,596 to 2,791 | 0,9906 |
| 1A vs. 2B | -0,6952 | -3,028 to 1,638 | 0,9841 |
| 1A vs. 3 | 1,179 | -1,026 to 3,383 | 0,7227 |
| 1A vs. 4 | 0,3867 | -1,867 to 2,640 | 0,9995 |
| 1A vs. 5 | 0,04952 | -2,229 to 2,328 | > 0,9999 |
| 1A vs. IVIg control | -0,8500 | -3,775 to 2,075 | 0,9863 |
| 1B vs. 2A | 0,8730 | 0,01563 to 1,730 | 0,0427 |
| 1B vs. 2B | -0,4201 | -1,589 to 0,7490 | 0,9549 |
| 1B vs. 3 | 1,454 | 0,5688 to 2,339 | < 0,0001 |
| 1B vs. 4 | 0,6617 | -0,3394 to 1,663 | 0,4640 |
| 1B vs. 5 | 0,3246 | -0,7322 to 1,381 | 0,9811 |
| 1B vs. IVIg control | -0,5749 | -2,691 to 1,541 | 0,9908 |
| 2A vs. 2B | -1,293 | -2,597 to 0,01112 | 0,0538 |
| 2A vs. 3 | 0,5810 | -0,4762 to 1,638 | 0,6939 |
| 2A vs. 4 | -0,2113 | -1,367 to 0,9448 | 0,9992 |
| 2A vs. 5 | -0,5484 | -1,753 to 0,6562 | 0,8562 |
| 2A vs. IVIg control | -1,448 | -3,641 to 0,7455 | 0,4657 |
| 2B vs. 3 | 1,874 | 0,5514 to 3,197 | 0,0006 |
| 2B vs. 4 | 1,082 | -0,3211 to 2,485 | 0,2635 |
| 2B vs. 5 | 0,7447 | -0,6985 to 2,188 | 0,7577 |
| 2B vs. IVIg control | -0,1548 | -2,488 to 2,178 | > 0,9999 |
| 3 vs. 4 | -0,7923 | -1,969 to 0,3846 | 0,4393 |
| 3 vs. 5 | -1,129 | -2,354 to 0,09515 | 0,0941 |
| 3 vs. IVIg control | -2,029 | -4,233 to 0,1755 | 0,0955 |
| 4 vs. 5 | -0,3371 | -1,648 to 0,9737 | 0,9934 |
| 4 vs. IVIg control | -1,237 | -3,490 to 1,017 | 0,6955 |
| 5 vs. IVIg control | -0,8995 | -3,178 to 1,379 | 0,9268 |

**Supplementary table 1.** Differences between IVIg preparations in respect of four glycosylation features for IgG1 and IgG2/3.

**IgG1 Galactosylation**

**IgG1 Sialylation**

| Tukey's multiple comparisons test | Mean Diff, | 95% CI of diff, | Adjusted  P Value |
| --- | --- | --- | --- |
|  |  |  |  |
| 1A vs. 1B | -0,08823 | -1,676 to 1,499 | > 0,9999 |
| 1A vs. 2A | -0,06417 | -1,710 to 1,582 | > 0,9999 |
| 1A vs. 2B | -0,4539 | -2,204 to 1,297 | 0,9930 |
| 1Avs. 3 | 0,6702 | -0,9839 to 2,324 | 0,9167 |
| 1A vs. 4 | -0,3610 | -2,052 to 1,330 | 0,9979 |
| 1A vs. 5 | -0,3081 | -2,018 to 1,402 | 0,9993 |
| 1A vs. IVIg control | -0,5000 | -2,694 to 1,694 | 0,9969 |
| 1B vs. 2A | 0,02406 | -0,6192 to 0,6673 | > 0,9999 |
| 1B vs. 2B | -0,3657 | -1,243 to 0,5115 | 0,9043 |
| 1B vs. 3 | 0,7584 | 0,09419 to 1,423 | 0,0134 |
| 1B vs. 4 | -0,2728 | -1,024 to 0,4784 | 0,9523 |
| 1B vs. 5 | -0,2199 | -1,013 to 0,5731 | 0,9896 |
| 1B vs. IVIg control | -0,4118 | -1,999 to 1,176 | 0,9930 |
| 2A vs. 2B | -0,3898 | -1,368 to 0,5888 | 0,9234 |
| 2A vs. 3 | 0,7343 | -0,05892 to 1,528 | 0,0917 |
| 2A vs. 4 | -0,2969 | -1,164 to 0,5705 | 0,9653 |
| 2A vs. 5 | -0,2439 | -1,148 to 0,6599 | 0,9911 |
| 2A vs. IVIg control | -0,4358 | -2,082 to 1,210 | 0,9921 |
| 2B vs. 3 | 1,124 | 0,1317 to 2,117 | 0,0147 |
| 2B vs. 4 | 0,09290 | -0,9597 to 1,146 | > 0,9999 |
| 2B vs. 5 | 0,1458 | -0,9370 to 1,229 | 0,9999 |
| 2B vs. IVIg control | -0,04606 | -1,797 to 1,704 | > 0,9999 |
| 3 vs. 4 | -1,031 | -1,914 to -0,1482 | 0,0103 |
| 3 vs. 5 | -0,9782 | -1,897 to -0,05945 | 0,0281 |
| 3 vs. IVIg control | -1,170 | -2,824 to 0,4839 | 0,3728 |
| 4 vs. 5 | 0,05295 | -0,9306 to 1,036 | > 0,9999 |
| 4 vs. IVIg control | -0,1390 | -1,830 to 1,552 | > 0,9999 |
| 5 vs. IVIg control | -0,1919 | -1,902 to 1,518 | > 0,9999 |

**IgG1 Fucosylation**

| Tukey's multiple comparisons test | Mean Diff, | 95% CI of diff, | Adjusted  P Value |
| --- | --- | --- | --- |
|  |  |  |  |
| 1A vs. 1B | 0,1365 | -0,4540 to 0,7269 | 0,9966 |
| 1A vs. 2A | -0,2150 | -0,8271 to 0,3971 | 0,9601 |
| 1A vs. 2B | -0,6158 | -1,267 to 0,03529 | 0,0783 |
| 1A vs. 3 | 0,4065 | -0,2087 to 1,022 | 0,4643 |
| 1A vs. 4 | -0,7110 | -1,340 to -0,08217 | 0,0150 |
| 1A vs. 5 | -0,4731 | -1,109 to 0,1628 | 0,3076 |
| 1A vs. IVIg control | 0,1500 | -0,6661 to 0,9661 | 0,9992 |
| 1B vs. 2A | -0,3515 | -0,5907 to -0,1122 | 0,0003 |
| 1B vs. 2B | -0,7522 | -1,078 to -0,4260 | < 0,0001 |
| 1B vs. 3 | 0,2701 | 0,02303 to 0,5171 | 0,0215 |
| 1B vs. 4 | -0,8475 | -1,127 to -0,5681 | < 0,0001 |
| 1B vs. 5 | -0,6096 | -0,9045 to -0,3146 | < 0,0001 |
| 1B vs. IVIg control | 0,01354 | -0,5769 to 0,6040 | > 0,9999 |
| 2A vs. 2B | -0,4008 | -0,7647 to -0,03681 | 0,0200 |
| 2A vs. 3 | 0,6215 | 0,3265 to 0,9165 | < 0,0001 |
| 2A vs. 4 | -0,4960 | -0,8186 to -0,1734 | 0,0001 |
| 2A vs. 5 | -0,2581 | -0,5942 to 0,07805 | 0,2687 |
| 2A vs. IVIg control | 0,3650 | -0,2471 to 0,9771 | 0,5985 |
| 2B vs. 3 | 1,022 | 0,6532 to 1,391 | < 0,0001 |
| 2B vs. 4 | -0,09528 | -0,4868 to 0,2962 | 0,9953 |
| 2B vs. 5 | 0,1427 | -0,2601 to 0,5454 | 0,9582 |
| 2B vs. IVIg control | 0,7658 | 0,1147 to 1,417 | 0,0095 |
| 3 vs. 4 | -1,118 | -1,446 to -0,7891 | < 0,0001 |
| 3 vs. 5 | -0,8796 | -1,221 to -0,5379 | < 0,0001 |
| 3 vs. IVIg control | -0,2565 | -0,8717 to 0,3587 | 0,9042 |
| 4 vs. 5 | 0,2379 | -0,1278 to 0,6037 | 0,4855 |
| 4 vs. IVIg control | 0,8610 | 0,2322 to 1,490 | 0,0011 |
| 5 vs. IVIg control | 0,6231 | -0,01282 to 1,259 | 0,0593 |

**IgG1 Bisecting GlcNAc**

| Tukey's multiple comparisons test | Mean Diff, | 95% CI of diff, | Adjusted  P Value |
| --- | --- | --- | --- |
|  |  |  |  |
| 1A vs. 1B | 0,03568 | -1,427 to 1,498 | > 0,9999 |
| 1A vs. 2A | 0,1246 | -1,392 to 1,641 | > 0,9999 |
| 1A vs. 2B | 1,590 | -0,02311 to 3,203 | 0,0565 |
| 1A vs. 3 | -0,9644 | -2,488 to 0,5596 | 0,5221 |
| 1A vs. 4 | 1,423 | -0,1352 to 2,981 | 0,1009 |
| 1A vs. 5 | 0,9912 | -0,5842 to 2,567 | 0,5297 |
| 1A vs. IVIg control | 0,003334 | -2,018 to 2,025 | > 0,9999 |
| 1B vs. 2A | 0,08891 | -0,5038 to 0,6816 | 0,9998 |
| 1B vs. 2B | 1,554 | 0,7458 to 2,362 | < 0,0001 |
| 1B vs. 3 | -1,000 | -1,612 to -0,3881 | < 0,0001 |
| 1B vs. 4 | 1,387 | 0,6949 to 2,079 | < 0,0001 |
| 1B vs. 5 | 0,9555 | 0,2249 to 1,686 | 0,0023 |
| 1B vs. IVIg control | -0,03234 | -1,495 to 1,430 | > 0,9999 |
| 2A vs. 2B | 1,465 | 0,5635 to 2,367 | < 0,0001 |
| 2A vs. 3 | -1,089 | -1,820 to -0,3581 | 0,0003 |
| 2A vs. 4 | 1,298 | 0,4990 to 2,097 | < 0,0001 |
| 2A vs. 5 | 0,8666 | 0,03389 to 1,699 | 0,0350 |
| 2A vs. IVIg control | -0,1213 | -1,638 to 1,395 | > 0,9999 |
| 2B vs. 3 | -2,554 | -3,468 to -1,640 | < 0,0001 |
| 2B vs. 4 | -0,1670 | -1,137 to 0,8029 | 0,9995 |
| 2B vs. 5 | -0,5985 | -1,596 to 0,3992 | 0,5910 |
| 2B vs. IVIg control | -1,586 | -3,199 to 0,02644 | 0,0575 |
| 3 vs. 4 | 2,387 | 1,574 to 3,201 | < 0,0001 |
| 3 vs. 5 | 1,956 | 1,109 to 2,802 | < 0,0001 |
| 3 vs. IVIg control | 0,9677 | -0,5562 to 2,492 | 0,5175 |
| 4 vs. 5 | -0,4315 | -1,338 to 0,4747 | 0,8251 |
| 4 vs. IVIg control | -1,419 | -2,977 to 0,1385 | 0,1025 |
| 5 vs. IVIg control | -0,9879 | -2,563 to 0,5875 | 0,5341 |

**IgG2/3 Galactosylation**

| Tukey's multiple comparisons test | Mean Diff, | 95% CI of diff, | Adjusted  P Value |
| --- | --- | --- | --- |
|  |  |  |  |
| 1A vs. 1B | 2,531 | -0,2913 to 5,354 | 0,1142 |
| 1A vs. 2A | 2,973 | 0,04736 to 5,899 | 0,0435 |
| 1A vs. 2B | 1,319 | -1,793 to 4,432 | 0,8963 |
| 1A vs. 3 | 3,314 | 0,3732 to 6,255 | 0,0156 |
| 1A vs. 4 | 1,553 | -1,453 to 4,559 | 0,7567 |
| 1A vs. 5 | 1,756 | -1,284 to 4,796 | 0,6372 |
| 1A vs. IVIg control | 1,633 | -2,268 to 5,535 | 0,9023 |
| 1B vs. 2A | 0,4421 | -0,7015 to 1,586 | 0,9341 |
| 1B vs. 2B | -1,212 | -2,771 to 0,3477 | 0,2545 |
| 1B vs. 3 | 0,7827 | -0,3981 to 1,964 | 0,4602 |
| 1B vs. 4 | -0,9783 | -2,314 to 0,3572 | 0,3274 |
| 1B vs. 5 | -0,7752 | -2,185 to 0,6345 | 0,6933 |
| 1B vs. IVIg control | -0,8979 | -3,720 to 1,925 | 0,9769 |
| 2A vs. 2B | -1,654 | -3,394 to 0,08581 | 0,0753 |
| 2A vs. 3 | 0,3406 | -1,070 to 1,751 | 0,9955 |
| 2A vs. 4 | -1,420 | -2,963 to 0,1217 | 0,0950 |
| 2A vs. 5 | -1,217 | -2,824 to 0,3895 | 0,2849 |
| 2A vs. IVIg control | -1,340 | -4,266 to 1,586 | 0,8523 |
| 2B vs. 3 | 1,995 | 0,2301 to 3,759 | 0,0150 |
| 2B vs. 4 | 0,2335 | -1,638 to 2,105 | > 0,9999 |
| 2B vs. 5 | 0,4366 | -1,489 to 2,362 | 0,9970 |
| 2B vs. IVIg control | 0,3139 | -2,798 to 3,426 | > 0,9999 |
| 3 vs. 4 | -1,761 | -3,331 to -0,1911 | 0,0164 |
| 3 vs. 5 | -1,558 | -3,192 to 0,07555 | 0,0734 |
| 3 vs. IVIg control | -1,681 | -4,621 to 1,260 | 0,6498 |
| 4 vs. 5 | 0,2030 | -1,546 to 1,952 | > 0,9999 |
| 4 vs. IVIg control | 0,08042 | -2,926 to 3,087 | > 0,9999 |
| 5 vs. IVIg control | -0,1226 | -3,162 to 2,917 | > 0,9999 |

**IgG2/3 Sialylation**

| Tukey's multiple comparisons test | Mean Diff, | 95% CI of diff, | Adjusted  P Value |
| --- | --- | --- | --- |
|  |  |  |  |
| 1A vs. 1B | 1,387 | -0,6033 to 3,378 | 0,3928 |
| 1A vs. 2A | 1,087 | -0,9767 to 3,150 | 0,7381 |
| 1A vs. 2B | 0,7103 | -1,484 to 2,905 | 0,9745 |
| 1A vs. 3 | 2,026 | -0,04799 to 4,100 | 0,0608 |
| 1A vs. 4 | 0,1829 | -1,937 to 2,303 | > 0,9999 |
| 1A vs. 5 | 0,6017 | -1,542 to 2,745 | 0,9888 |
| 1A vs. IVIg control | 0,7233 | -2,028 to 3,474 | 0,9924 |
| 1B vs. 2A | -0,3005 | -1,107 to 0,5060 | 0,9455 |
| 1B vs. 2B | -0,6768 | -1,777 to 0,4229 | 0,5585 |
| 1B vs. 3 | 0,6386 | -0,1941 to 1,471 | 0,2701 |
| 1B vs. 4 | -1,204 | -2,146 to -0,2624 | 0,0032 |
| 1B vs. 5 | -0,7855 | -1,780 to 0,2087 | 0,2352 |
| 1B vs. IVIg control | -0,6638 | -2,654 to 1,327 | 0,9699 |
| 2A vs. 2B | -0,3764 | -1,603 to 0,8505 | 0,9812 |
| 2A vs. 3 | 0,9391 | -0,05544 to 1,934 | 0,0792 |
| 2A vs. 4 | -0,9037 | -1,991 to 0,1837 | 0,1812 |
| 2A vs. 5 | -0,4850 | -1,618 to 0,6481 | 0,8915 |
| 2A vs. IVIg control | -0,3633 | -2,427 to 1,700 | 0,9994 |
| 2B vs. 3 | 1,315 | 0,07120 to 2,560 | 0,0301 |
| 2B vs. 4 | -0,5274 | -1,847 to 0,7923 | 0,9222 |
| 2B vs. 5 | -0,1086 | -1,466 to 1,249 | > 0,9999 |
| 2B vs. IVIg control | 0,01303 | -2,182 to 2,208 | > 0,9999 |
| 3 vs. 4 | -1,843 | -2,950 to -0,7358 | < 0,0001 |
| 3 vs. 5 | -1,424 | -2,576 to -0,2721 | 0,0050 |
| 3 vs. IVIg control | -1,302 | -3,376 to 0,7713 | 0,5321 |
| 4 vs. 5 | 0,4187 | -0,8143 to 1,652 | 0,9668 |
| 4 vs. IVIg control | 0,5404 | -1,579 to 2,660 | 0,9937 |
| 5 vs. IVIg control | 0,1217 | -2,022 to 2,265 | > 0,9999 |

**IgG2/3 Bisecting GlcNAc**

| Tukey's multiple comparisons test | Mean Diff, | 95% CI of diff, | Adjusted  P Value |
| --- | --- | --- | --- |
|  |  |  |  |
| 1A vs. 1B | -0,09177 | -0,9547 to 0,7712 | > 0,9999 |
| 1A vs. 2A | -0,2137 | -1,108 to 0,6808 | 0,9958 |
| 1A vs. 2B | 0,5876 | -0,3639 to 1,539 | 0,5541 |
| 1A vs. 3 | -0,5365 | -1,436 to 0,3626 | 0,5976 |
| 1A vs. 4 | 0,5598 | -0,3593 to 1,479 | 0,5718 |
| 1A vs. 5 | 0,3681 | -0,5613 to 1,297 | 0,9255 |
| 1A vs. IVIg control | -0,03000 | -1,223 to 1,163 | > 0,9999 |
| 1B vs. 2A | -0,1220 | -0,4716 to 0,2277 | 0,9615 |
| 1B vs. 2B | 0,6793 | 0,2025 to 1,156 | 0,0006 |
| 1B vs. 3 | -0,4447 | -0,8058 to -0,08371 | 0,0053 |
| 1B vs. 4 | 0,6516 | 0,2432 to 1,060 | < 0,0001 |
| 1B vs. 5 | 0,4599 | 0,02885 to 0,8909 | 0,0276 |
| 1B vs. IVIg control | 0,06177 | -0,8012 to 0,9247 | > 0,9999 |
| 2A vs. 2B | 0,8013 | 0,2694 to 1,333 | 0,0002 |
| 2A vs. 3 | -0,3228 | -0,7539 to 0,1084 | 0,2999 |
| 2A vs. 4 | 0,7735 | 0,3021 to 1,245 | < 0,0001 |
| 2A vs. 5 | 0,5818 | 0,09058 to 1,073 | 0,0087 |
| 2A vs. IVIg control | 0,1837 | -0,7108 to 1,078 | 0,9984 |
| 2B vs. 3 | -1,124 | -1,664 to -0,5846 | < 0,0001 |
| 2B vs. 4 | -0,02778 | -0,6000 to 0,5444 | > 0,9999 |
| 2B vs. 5 | -0,2195 | -0,8081 to 0,3691 | 0,9452 |
| 2B vs. IVIg control | -0,6176 | -1,569 to 0,3339 | 0,4884 |
| 3 vs. 4 | 1,096 | 0,6163 to 1,576 | < 0,0001 |
| 3 vs. 5 | 0,9046 | 0,4052 to 1,404 | < 0,0001 |
| 3 vs. IVIg control | 0,5065 | -0,3926 to 1,406 | 0,6664 |
| 4 vs. 5 | -0,1917 | -0,7263 to 0,3429 | 0,9554 |
| 4 vs. IVIg control | -0,5898 | -1,509 to 0,3293 | 0,5036 |
| 5 vs. IVIg control | -0,3981 | -1,327 to 0,5313 | 0,8912 |
