# Supplementary material for: Comparison of Fc N-Glycosylation of Pharmaceutical Products of Intravenous Immunoglobulin G
Source: PLoS One. 2015 Oct 12;10(10):e0139828. doi: 10.1371/journal.pone.0139828 (PMC4601728; doi:10.1371/journal.pone.0139828)
Supplement: S2 Table — (DOCX) [file pone.0139828.s004.docx]

00A07AX11

95D04AB11A

LE08D007AJ

LE12J225AL

LE12J313AG

LE12J360AC

LE12J388AB

LE12L097AB

LE12L188AD (1)

LE12L188AD (2)

LE12L188AD (3)

LE12L308AW

LE12L315AD

LE12L315AZ

LE12L346AM

LE12L360BD

LE12L362AN

LE12L378AC

LE12M002AE

LE12M013AW

LE12M035AF

LE12M035AM

LE12M052BA

LE12M052BK

LE12M052BZ

LE12M063AG

LE12M063AL

LE12M065AP

LE12M066AK

LE12M066AS

LE12M083AC

LE12M092AD

LE12M096AL

LE12M096AZ

LE12M118AM

LE12M122AG

LE12M139AP

LE12M140AH

LE12M164AW

LE12M173AB

LE12M178AB

LE12M200AJ

LE12M201AG

LE12M211AZ

LE12M233AK

LE12M241AF

LE12M247AM

LE12M248AF

LE12M257AD

LE12M272AP

LE12M282AF

LE12M301AD

LE12M311AB

LE12M311AE

LE12M313AC

LE12M320AM

LE12MA41AD

LE12MA52AG

LE12MC11AC

LE12MC48AG

LE12N010AC

LE12N035AE

LE12N038AD

LE12N046AD

LE12N047AF

LE12N049AJ

LE12N066AH

LE12N073AL

LE12N073AS

ZLB04_003 001

ZLB04_003 002

ZLB04_003 003

ZLB04_003 004

ZLB04_003 005

ZLB04_003 006

ZLB04_003 007

ZLB04_003 008

ZLB04_003 009

ZLB04_003 010

ZLB04_003 011

ZLB04_003 012

ZLB04_003 013

ZLB04_003 014

ZLB04_003 015

ZLB04_003 016

ZLB04_003 101

ZLB04_003 102

ZLB04_003 103

ZLB04_003 104

ZLB04_003 105

ZLB04_003 106

ZLB04_003 107

ZLB04_003 201

4324000018

4324100034

4324100036

4324100040

4324100041

4324100042

4324100043

4324200045

4324200046

4324200047

4324200048

09A28H460A

09D07H462A

09D22H464A

10A27H465A

10D21H464A

10H09H462A

10L01H465A

11A21H466A

11A26H466A

11I01H464A

11J12H466A

11J19H464A

11K30H466A

12H10H462A

12I07H465A

12J18H465A

12K07H465A

12K21H465A

12L11H466A

13A08H466A

13A09H466A

13E15H466A

IBG2ASAT1

IBGJ2C9CA1

IBGJ2GBC1

IBGJ2HNHP1

IBGJ3RLRM1

IBGK0RPRR1

IBGK2AAAB1

IBGK2DZE11

IBGK2I8J61

IBGK2KBKC1

IBGL1JRJS1

IBGL1N3N41

IBGL2A2A31

IBGL2B2B31

IBGL2IBIC1

IBGL2KVL71

A207B8536

A221A853D

A222B8533

B219A8542

B222A8541

B227B8542

C150A8531

C216A8545

C216B8542

C217A8543

C218A8541

C221A8542

C222B8545

C227B8531
